# Ionized or Total Magnesium levels, what should we measure in critical ill patients?

**DOI:** 10.37825/2239-9747.1015

**Published:** 2020-10-01

**Authors:** G Scarpati, D Baldassarre, F Oliva, G Pascale, O Piazza

**Affiliations:** University of Salerno

**Keywords:** Ionised Magnesium, Preoperative Medicine, ICU, Dysmagnesemia

## Abstract

Monitoring and measuring magnesium (Mg) values are essential to prevent the development of numerous complications in perioperative medicine and critically ill patients. Although previous studies suggest that measuring free ionized magnesium (iMg) is more useful for estimating Mg status, clinicians currently rely on measurement of total serum magnesium to determine if supplemental magnesium is needed. In this review, we analyzed the recent literature to decide whether it is better to measure ionized serum Mg or total serum Mg when assessing magnesium status, whether iMg predicts clinical outcome, and what are the difficulties in measuring serum iMg levels in intensive care patients and perioperative medicine.

## I. IONIZED VS TOTAL MAGNESIUM LEVELS IN INTENSIVE CARE PATIENTS

Ionized magnesium (iMg) constitutes 50% of the total magnesemia and represents the electrophysiologically active portion. 10% of magnesium in the blood is complexed with anions, and the remaining 30–40% is bound to albumin [1]. Hypomagnesaemia occurs postoperatively in about 60%, in 65% of patients admitted to medical intensive care (ICU) and in about 90% of surgical patients [2]. Although total Mg and ionized Mg are generally related in subjects in good health, the literature on Mg supplementation has been disparate [3–5]. iMg is rarely measured in a clinical setting [6], perhaps because it requires specialized equipment for measuring iMg on whole blood. Additionally, iMg values may be subject to individual factor interference. According Rooney M et al [4], who studied 59 relatively healthy individuals aged > 55 years and with no prior history of cardiovascular disease, randomized 1:1 to 400 mg/day of oral Mg (in the form of Mg oxide) or lactose placebo for 10 weeks, the baseline concentrations of iMg and tMg were modestly and positively associated. Very few studies have evaluated the relation between tMg and iMg in patients admitted to intensive care. Cardiovascular dysfunction and systemic inflammatory response syndrome in ICU patients are closely related to hypomagnesaemia [2]. Dysmagnesaemia is also common in critically ill patients and has been shown Table 1. Causes of hypomagnesemia in ICU patients. to be associated with a higher mortality rate and longer ventilatory support [7–9]. Also, several studies report a correlattion of dysmagnesemia with increased mortality, length of stay, and morbidity in ICU patients [10–14]. Nonetheless, hypomagnesemia is frequently underdiagnosed. Hypomagnesemia is present in approximately 70% of adult patients admitted to the ICU. Gastrointestinal disturbances and renal loss of Mg are the main causes of hypomagnesaemia in critically ill patients [15] (Table 1) and they are a frequent cause of secondary hypokalemia and hypocalcemia leading to severe neuromuscular and cardiovascular clinical manifestations (Table 2).

In particular, ICU patients receive different combinations of drugs and may have a reduced ability to eliminate the drugs due to the reduced kidney and/or liver function that could influence Mg homeostasis [15].

Finally, diabetic ketoacidosis, starvation or alcoholism causing metabolic acidosis result in the renal loss of Mg. Del Gobbo et al. demonstrate that for every 0.49 mg/dL (0.2 mmol/L) serum Mg reduction, there is a 30% increase in cardiovascular diseases [16]. Furthermore, other studies have reported a higher incidence of sepsis in ICU patients with hypomagnesaemia [10,11,17].

The relationship between hypomagnesaemia and mortality rate varies widely in the literature. Chernow et al. [18], Rubeiz et al. [19] and Safavi and Honarmand [20] found in their studies a higher mortality rate in patients with hypomagnesaemia than in normomagnesemic patients. While Guérin et al. [21] noted a higher ICU mortality rate among hypermagnesaemic patients with no difference in mortality between hypomagnesaemic and normomagnesemic patients. Finally, other studies [22,23] observed up to 3 times higher mortality rates in patients who develop ionized hypomagnesaemia while staying in the ICU. The average hospital mortality in all the studies was 24.3%. Hypermagnesemia is present in 5% to 10% of critically ill patients [24]. Table 2 shows the clinical conditions associated with hypo- and hypermagnesemia.

Several studies demonstrated that iMg is the only marker that specifically identifies patients with dysmagnesemia [25,26]. Besides, iMg monitoring of patients treated with Mg sulfate improves clinical outcomes. It can reduce the length of stay, and iMg monitoring reduces the risk of Mg toxicity in patients treated for Mg deficiency [26]. In critically ill patients, the correlation between iMg and total Mg has been demonstrated to be poor [27,28]. In fact, 69%–85% of critically ill patients with low total Mg results have been found to have normal iMg levels. Correcting Mg deficiency based on rapid, real-time measurement of iMg allows for a more accurate titration of Mg sulfate, but iMg must be directly measured; it cannot be calculated.

Yeh et Al. noted that using total Mg in clinical decision making can lead to over-treating patients with Mg sulfate and unnecessary repeat testing [27].

iMg is also vital for the intracellular regulation and transport of Na, K, and iCa [12]. So, rapid serial measurement of iMg is crucial for the management of electrolyte deficiencies [29]. Hypomagnesaemia and hypocalcemia are very common (up to 40%) in patients with other electrolyte abnormalities [30], demonstrating that iMg, Na, K, and Ca should always be tested together for proper electrolyte near-normal. In fact, it was observed that the hypokalemia and hypocalcemia did not improve on intravenous K and Ca administration alone, it was only after Mg supplementation, the blood levels of Na, K and Ca reached the near-normal levels [31]. Treatment of magnesium deficiency in hypomagnesaemia through intravenous administration requires a concentration of MgSO4 20% or less with a rate of injection not exceeding 1,5ml/minute of a 10% solution or its equivalent. For severe magnesium deficiency, about 5g/liter of infusion solution intravenously over 3 hours should be recommended. Dosage should be reduced in renal impairment. Plasma magnesium concentrations should be monitored throughout therapy. For a summary of clinical studies evaluating the role and dosing of magnesium sulfate (MgSO4) in critically ill patients please read Panahi et Al. [31].

## II. IONIZED VS TOTAL MAGNESIUM LEVELS IN THE PERIOPERATIVE SETTING

The interest in magnesium and its biologically active form continues to be attentive despite conflicting data of the effect of magnesium supplementation on preventing perioperative adverse events or predicting health outcomes in surgical patients. A state of perioperative hypomagnesemia, not present before surgery, has been reported [32,33], considering the total serum magnesium easily measurable. The available data on the possible benefit of the magnesium supplementation in the perioperative period concern different aspects.

The greatest attention has been pointed to the prevention of arrhythmias in high-risk patients undergoing cardiac surgery. A recent meta-analysis is supporting the data published in previous years on the role of prophylactic magnesium supplementation in the prevention of postoperative atrial fibrillation (POAF) in patients undergoing cardiac surgery [33], especially when magnesium is given in the postoperative period [34]. Perioperative hypomagnesemia should have a crucial influence on cardiac adverse events after surgery [35] particularly on POAF, described as the most common cardiac complication occurred in cardiothoracic surgery [36]. The reported data on cardiac surgery patients seem to be faulty by many confounding factors. Population recruited in these search patterns are often high-risk patients or with morbid conditions that can alter magnesium homeostasis, consequently, the total circulating magnesium. In the same way, they often require additional intraoperative therapies and different surgical techniques. Furthermore, the ubiquitous role that magnesium has in biological functions makes it difficult to identify all the interconnections between magnesium homeostasis and the causes of perioperative adverse events magnesium correlated. Although further investigation is needed and the mechanisms involved are unclear, magnesium plays a role in the incidence of cardiac complications and perioperative mortality [37]. The work of Whittaker et al. [37] pointed out that perioperative changes in serum magnesium are independently predictive for 30-day mortality and cardiac morbidity on patients undergoing unplanned vascular surgery. The relevance of the data is considerable, but the authors do not address the issue of the physiopathology of surgical related hypomagnesemia, nor examine the effects of magnesium supplements on patient outcomes suggesting further high-quality research to probe the effects of the biologically active form of magnesium, its measurement and its usefulness in the perioperative setting.

The intravenous administration of magnesium has proven to be safe in any case [33,38], and it has already been used as an effective therapy in the torsade de pointes [39]. The effectiveness of magnesium supplementation in the prevention of postoperative supraventricular arrhythmias seems to be the same for thoracic surgery [40]. Considering the already approved uses of magnesium, some international companies do not underestimate the results obtained until now in preventing perioperative adverse events especially in the cardiothoracic and vascular surgery and recommend the prophylactic use of intravenous magnesium for high-risk patients [41].

Less consistent results relate to the effect of additional magnesium in the perioperative non-cardiac setting. A recent meta-analysis reported magnesium prophylaxis’s ineffectiveness in reducing the incidence of arrhythmias after non-cardiac surgery and no impact on mortality [42]. It is right to underline that patients included in this meta-analysis are underwent different types of non-cardiac surgery with significant heterogeneity in the trials recruited. The efficacy of additional serum magnesium to prevent intra and postoperative arrhythmic events is often compared, as in this case, with antiarrhythmics or vasoactive drugs steadily administered for the pre-existing pathology in the general population candidate for surgery. Do not measure the ionized magnesium to be replenished and, therefore, the useful proportion of serum magnesium, it may not be helpful but, on the contrary, can create confusion and lack of reliability in the data. Several drugs commonly used, including diuretics and proton-pump inhibitors, interfere with the magnesium homeostasis. There are many different factors related to the patient or pre-existing diseases that can affect the absorption, metabolism, and excretion of magnesium [43].

In the same way, it can be misleading to consider only the dose of magnesium administered for all patients despite the promising results, without considering the sufficient serum concentrations achieved. According to recent work, intravenous magnesium at a dose of 50 mg/Kg is associated with significantly modulated hemodynamic response during laparoscopic surgery, reducing diastolic pressure and heart rate [44]. The reason for choosing a specific intravenous magnesium dosage, as reported in the meta-analysis in question, which includes 4 RCTs, remains unknown.

Some studies evaluated the achievement of stable magnesium levels after bolus intravenous administration, how it is usually used, or followed by continuous infusion that is demonstrated the same effective levels [45]. The oral route has been suggested in prophylactic therapy to prevent postoperative arrhythmias [46], but these investigations have aimed at measurement on total magnesium and not ionized bioactive free form [47].

Although no guidelines exist for the prophylactic use of magnesium in non-cardiac surgery and the research procedures implemented to achieve such important results are still unclear, a recent survey shows that, about almost a thousand doctors interviewed, 35% regularly use magnesium in anesthesia with a variable dosage based on personal choices [48]. Perioperative use of magnesium is also reported in the literature as an analgesic adjuvant during anesthesia or administered to reduce the demand of anesthetic drugs. Used for the pain control after laparoscopic cholecystectomy, intravenous magnesium is effective in reducing early pain within 24 hours and the requirement of anesthetic drugs [49]. This meta-analysis, with low heterogeneity and favorable outcome on pain control, have referred intravenous doses of magnesium from 7.5 mg/Kg to 50 mg/Kg or in association with continuous infusion among all trials considered. The same efficacy with various doses is reported in a systematic review investigating the effects of the supplement of magnesium in orthopedic surgery. The reported dose varied among studies considered but all within 50 mg/kg considering the maximum safe dose [50].

Other useful results on the control of postoperative pain emerge from the use of magnesium combined with local epidural anesthetic in different types of non-cardiac surgery in another series of trials evaluated [51]. Even in this case, there is a large dosage range of magnesium without serum detection. Ionized Magnesium has been forgotten once again. Considering the rule of magnesium as a non-competitive antagonist of the *N-methyl*-d-aspartate (NMDA) receptor and its possible action on nociceptive modulation in the spinal cord [52], for several years, has been attempted to demonstrate its effectiveness on the extension of the action of intrathecal local anesthetics and opioids. Summarizing the data of several RCTs, Morrison et al. suggest the advantage of adding intrathecal magnesium to lipophilic opioids with or without local anesthetics to prolong the duration of spinal anesthesia in all types of surgery except for the obstetric surgery, encouraging the implementations of other trials [53]. An interesting scientific debate has emerged about the safety and the right methods to perform a trial without neglecting the possible neurotoxicity of the intrathecal magnesium. While some authors assert safe and efficacious intrathecal magnesium dosages from 50 to 100 mg/kg without reported complications [54], others consider it possibly dangerous and thinking there is not currently licensed for intrathecal administration [55]. We add that to avoid side effects, safety levels can be established when they are accurately measured in the blood regardless of the administration route. The evidence that magnesium is an adjuvant in the control of postoperative pain emerges forcefully. The reduction of the opioid request and the prolonged analgesia are also obtained by the administration of the magnesium when associated with the local anesthetics in the peripheral blocks [56]. Whatever is the route of administration, intravenous, peridural, intrathecal, or peripheral, the level of adequate active magnesium is not detected, the limit for the onset of toxicity has not yet been proven and the difficulty of performing reliable trials remains.

## III. DISCUSSION

**Figure 1:**
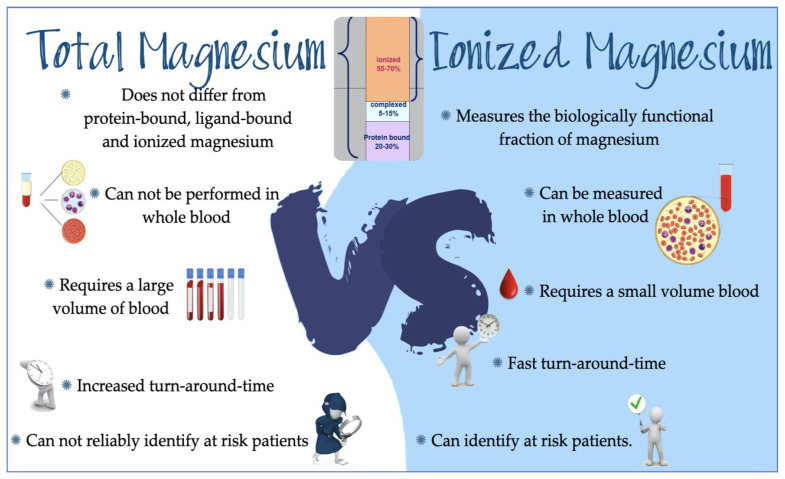
Total Magnesium vs Ionized Magnesium. Why in our opinion we should measure ionized magnesium.

Ionized serum magnesium is the only marker that specifically identifies critically ill patients with impaired magnesium status and surgical patients that require a reduction of perioperative complications due to magnesium deficiency. Magnesium represents the second cation present in the intracellular environment and the fourth most abundant in the entire body. It plays a crucial task in the synthesis of nucleic acids and proteins, in the activity of hundreds of enzymes and is essential in neuromuscular contractility and in generating potential cardiac action [57]. Monitoring circulating ionized magnesium is to be preferred as a measure of bioavailability after supplementation of magnesium compared with total magnesium, and it reduces the risk of Mg toxicity in patients treated for Mg deficiency. To increase the reliability of measurement iMg must be directly measured and it cannot be calculated. Even though critically ill patients are the most exposed to dysmagnesemia, iMg is not frequently measured in this clinical asset. And is not frequently measured in operating rooms either. Specialized equipment is needed for iMg measurement and it requires an immediate whole blood test. Detected iMg corresponds to the portion of magnesium biologically functional in contrast with total magnesium which includes protein-bound, ligand-bound, and the ionized magnesium. In our opinion iMg is the preferable clinical biomarker of magnesium status in critically ill patients that can avoid overtreatment and identify patients with true dysmagnesemia with a higher risk of death (Figure 1)

The data regarding the use of magnesium sulphate intravenously is encouraging in surgical patients, especially in cardiothoracic surgery, in order to prevent cardiac adverse events, improve outcomes, and reduce the perioperative mortality rate. The most significant results have been detected in preventing supraventricular arrhythmias in high-risk patients undergoing cardiac surgery. Perioperative hypomagnesemia is described mostly in cardiac surgical patients. They are commonly high-risk patients with pre-existing pathological conditions or developed in perioperative period that can alter magnesium homeostasis. They often require additional intraoperative drugs and different surgical techniques or extracorporeal circulation that interfere with the magnesium homeostasis. While some scientific societies suggest the use of magnesium in cardiothoracic surgery, no guidelines exist for the prophylactic use of magnesium sulphate in non-cardiac surgery. The correlation between total magnesium and ionized magnesium in these clinical assets has not been tested and most studies do not use iMg measurements to correlate the addition of magnesium to the improvement of outcomes.

Despite the real benefit of magnesium replacement in reduced mortality, the proven under-diagnosed hypomagnesemia in critically ill and surgical patients, too few studies are investing in the trials that well-characterized the optimal concentrations of iMg that imply health outcomes and avoid toxicity effects.

Different attempts to standardize the dose to be administrated, in literature, draw from the employment of magnesium in perioperative pain management. We reported intravenous doses of magnesium sulfate ranging from 7.5 mg/Kg to 50 mg/Kg in different RCTs and from 50 mg/Kg to 100 mg/Kg dose of intrathecal magnesium sulfate without a specific explanation when choosing one or another. And the same goes for the epidural route with ranges that can reach 500 mg of magnesium sulfate in continuous infusion or ultrasound-guided TAP-block that has a significant effect on postoperative pain control with ranges half as significant. The role of magnesium addition using different administration routes is widely proven to be effective in extending duration of analgesia, reducing postoperative analgesic drug necessity, and positively influencing hemodynamic responses during laparoscopic surgery. There are still no indications or recommendations on the use of magnesium in perioperative pain management and the safe threshold has yet to be established. None of the reported trials have detected circulating magnesium or iMg before and after administration nor has research, despite the positive outcomes, identified the correct and effective dose. Regularly measuring ionized magnesium levels is likely to play an essential role in defining the average effective dose that, in addition, would help preventing, mainly when using different administration routes, overdosage of magnesium. The clinical ranges reported by literature regarding total serum magnesium in the adult range from 0,7 to 1 mmol/L with minor variations and often suffer further deviations ranged between 0,75 and 0,95 [58]. It is widely acknowledged that the reference ranges proposed and currently in use for total magnesium originated from many studies regarding magnesium detection in healthy group samples with public health consensus. Total serum Mg is still considered by many authors as a good biomarker of magnesium status but only because it is the only test currently used in clinical practice at a reasonable cost and with standardized methodologies. Accredited reference ranges derive from studies that had the possibility to measure total serum magnesium, urinary magnesium and fractional excretion with procedures extensively used in clinical laboratories and therefore easily available for clinical research. Variations in the concentration of total serum magnesium, easily detectable, has been correlated with the extracellular fraction of magnesium considered in equilibrium among different body compartments involved in magnesium homeostasis. But total magnesium measurement in serum does not reflect intracellular concentrations or the available unbound fraction, moreover it doesn’t take into account the percentage of Mg bounded to proteins, to other molecules or water and doesn’t consider the dynamic flow of Mg throughout different body compartments in order to maintain homeostasis. Trials concerning correlation between clinical outcomes and magnesium status on ill patients with different pathologies and organ failures are not clear because of the large variability of different analytical methods used to conduct them [59]. As a matter of fact, variations in total magnesium that deviate from the validated ranges may not correspond to the bioavailability of the active form of magnesium and a quantity within the range may misleadingly indicate a erroneous bioavailability of active form. With values within or even above range there is a true possibility that individuals result already deficient in biologically active magnesium with a latent deficiency [58]. Why not measure the active form directly then? (Table 3) Reference values emerged in literature for ionized Mg are ranged from 0.54 to 0.67 mM/L with an average interval of only 130 micromolar and a fraction of ionized Mg that can exceed 70% of the total circulating magnesium. However, the evidenced-based data is still poor in order to obtain unanimous approval [60]. Ionized magnesium refers to free magnesium ions and their detection requires laboratory techniques other than those for the total Mg. The availability of magnesium ionselective electrode and specific sensor technology linked with commercial blood analyzers assures more reliable measurement of ionized magnesium in a clinical setting [61]. This allows specific data collection in clinical practice directly on patients taking into account all those conditions that can alter magnesium levels as: independent factors, interferences with intake, absorption, losses and excretion. This procedure will surely produce adequate information in order to assume reference levels based on the evidence. iMg can be detected in whole blood minimizing waiting time compared to the detection of serum samples of tMg. The measurement can be influenced by the presence of other ions especially calcium, by temperature, by pH or dilution of the sample [62], corrected by the calibration of the blood analyzer and the exact sampling carried out by the operators. The infrequent detection of iMg in clinical practice seems to draw from a lack of standardization of diagnostic reference levels rather than high costs. Moreover, there are no guidelines or recommendations regarding therapeutic doses. No data is available regarding the relation between total magnesium concentrations, the most surveyed, and ionized magnesium before and after the additional administration of sulphate magnesium. And no data is available regarding its proportional increment after reintegration. There is still no relation between the amount of i.v. sulphate magnesium administration, or other administration routes, in order to obtain reference levels of proposed iMg. It is yet to be clarified if such reference levels correspond, in clinical practice, to the effective concentrations of ionized magnesium in order to obtain the clinical results described in literature and above reported. Further and more specific research is essential to relate the detected values of ionized magnesium and its precise quantity to restore in order to optimize magnesium levels.

## IV. CONCLUSIONS

Hypomagnesemia (and Hyper-) are present in several clinical manifestations, including cardiovascular indication and sepsis. Accurate and routinely measuring ionized magnesium in critically ill patients is necessary to replace the active physiological part. Measure and monitor the biologically active form of magnesium and establish an adequate circulating threshold is still a current challenge. The specialized equipment required to measure the biologically active way instead of circulating total magnesium does the research demanding work. Most studies do not consider serum magnesium measurement at all but only after supplementation in the surgical patients. The inclusion of iMg in the critical care profile enables rapid assessment of the physiologically active and clinically relevant magnesium fraction. Dosage, administration route, and optimal timing in the perioperative setting should be clarified concerning the measured levels accurately when it is reasonable to give magnesium supplements to achieved clinical benefits. The infrequent detection of iMg in clinical practice is due to a lack of standardization of diagnostic reference levels rather than to high costs. No data is available regarding the relation between total magnesium concentrations and ionized magnesium before and after the additional administration of sulphate magnesium, and no data is available regarding iMg proportional increment after reintegration. To this purpose, a large clinical trial, in different clinical setting, is necessary.

## Figures and Tables

**Table 1:** Causes of hypomagnesemia in ICU patients.

Gastrointestinal disturbances
Nasogastric suction
Malabsorption syndrome
Bowel resection
Diarrhea
Malnutrition
Pancreatitis

**Renal loss of Mg**

Chronic parenteral fluid therapy
Osmotic diuresis
Hypercalcemia
Alcohol
Drugs
Metabolic acidosis
Renal diseases

**Table 2:** Clinical manifestations associated with hypomagnesemia and hypermagnesemia.

*Hypomagnesemia*	*Hypermagnesemia*
**Cardiovascular abnormalities**	**Cardiovascular abnormalities**

Atrial and ventricular arrhythmias	Heart Block
Hypertension	Hypotension
Cardiac insufficiency	Cardiac arrest
Coronary vasospasm	Bradycardia
Heart failure and sudden death	

**Neuromuscular abnormalities**	**Neuromuscular abnormalities**

Skeletal and respiratory muscle weakness	Respiratory distress
Tetany	Paralysis
Convulsions	Lethargy

**Metabolic abnormalities**	**Gastrointestinal abnormalities**

Hypokalemia	Nausea
Hypocalcemia Insulin resistance Impaired glucose tollerance	Vomiting

**General manifestations**	**Hemostatic abnormalities**

Weakness	Decreased thrombin generation
Anorexia	Decreased platelet aggregation
Depression Migraine	

**Table 3:** Difficulties in measuring serum iMg levels.

Difficulties in measuring serum iMg levels
Evidence-based data is still poor to gain unanimous approval on the iMg range [60]
Ionized magnesium refers to free magnesium ions and their detection requires laboratory techniques other than those for the total Mg.[61]
The measurement can be influenced by the presence of other ions especially calcium, by temperature, by pH or dilution of the sample [62]
Lack of standardization of diagnostic reference levels rather than high costs
